# Mercury and Mercury-Containing Preparations: History of Use, Clinical Applications, Pharmacology, Toxicology, and Pharmacokinetics in Traditional Chinese Medicine

**DOI:** 10.3389/fphar.2022.807807

**Published:** 2022-03-02

**Authors:** Meiling Zhao, Yi Li, Zhang Wang

**Affiliations:** ^1^ College of Pharmacy, Chengdu University of Traditional Chinese Medicine, Chengdu, China; ^2^ College of Ethnomedicine, Chengdu University of Traditional Chinese Medicine, Chengdu, China

**Keywords:** mercury, mercury-containing preparations, application history, clinical application, pharmacology, toxicology, pharmacokinetics

## Abstract

Historically, mercury and mercury-containing preparations have been widely used in traditional Chinese medicine and applied in many clinical practices mainly in the form of mercury sulfides. The clinical application, toxicity manifestations, and symptoms of these preparations largely depend on the route of administration and the dosage form. Commonly used mercury-containing medicinal materials and preparations in traditional Chinese medicine include Cinnabar, an excellent medicine for tranquilizing the nerves; Hongsheng Dan and Baijiang Dan, which have antibacterial, anti-inflammatory, promotion of tissue repair and regeneration and other pharmacological effects. Tibetan medicine commonly uses Zaotai and Qishiwei Zhenzhu pills, which have pharmacological effects such as sedation, anti-inflammatory, anti-convulsant, and improvement of cerebral apoplexy. Menggen Wusu Shibawei pills, commonly used in Mongolian traditional medicine, have the muscle growth and astringent effects. In India and Europe, mercury is often used for treating syphilis. This article summarizes the history, clinical application, pharmacology, toxicology, and pharmacokinetics of mercury and mercury-containing preparations in traditional medicines. In terms of clinical application, it provides suggestions for the rational use and safety of mercury-containing drugs in clinical practices and in public health issues. It will further provide a reference for formulation strategies related to mercury risk assessment and management.

**GRAPHICAL ABSTRACT:**
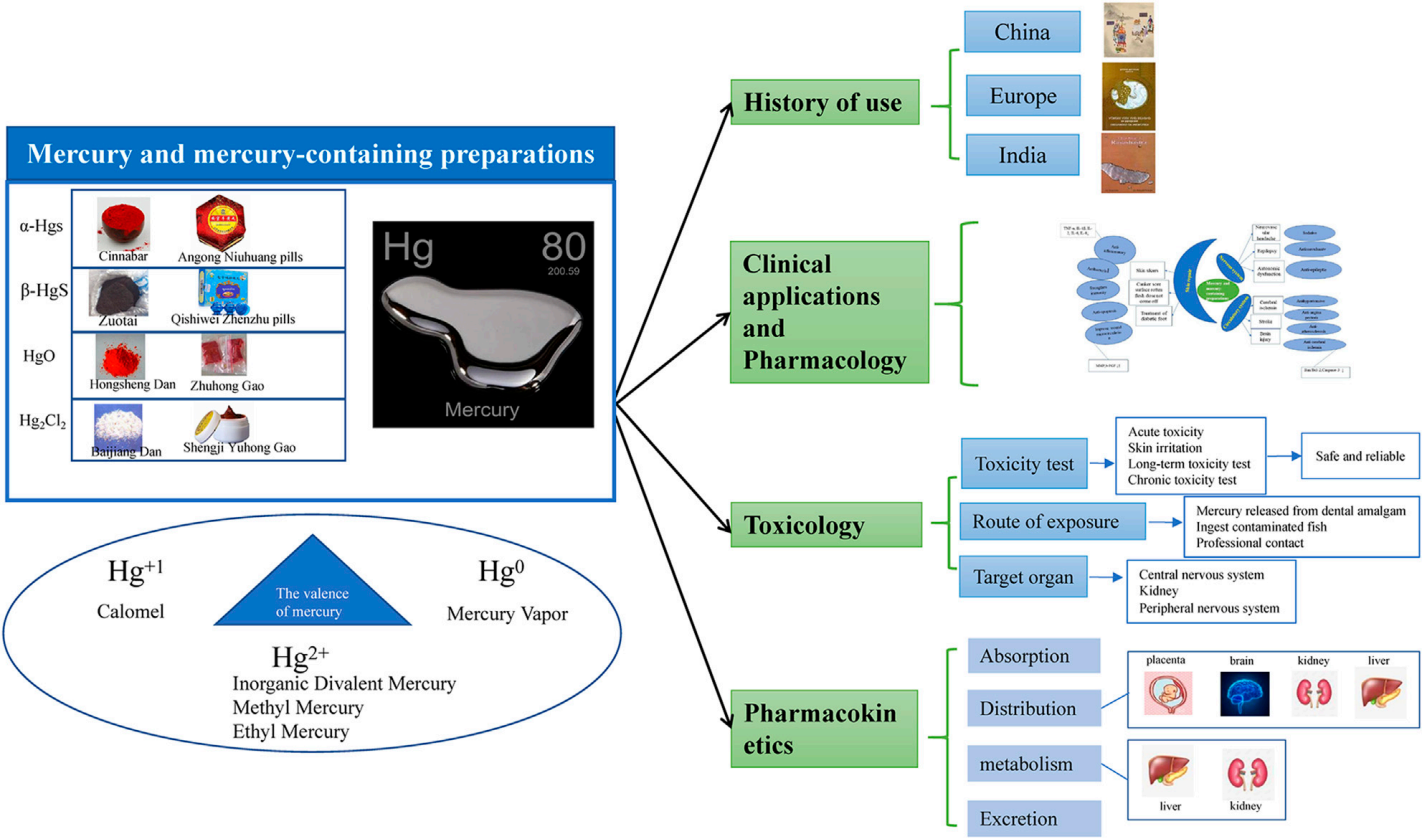


## Introduction

For a long time, mercury has been considered an almost magical substance, and it has been used for commercial and medical purposes to eradicate the most serious diseases. The medical use of mercury (liquid metal mercury) is well documented in medical and alchemy monographs from ancient Greece, India, Persia, Arabia, and China. Currently, mercury still plays an important role in traditional medicines in Asia and continues to be used in traditional Chinese medicine, Tibetan medicine, Ayurveda, Unani, and Siddha. Mercury has been used in traditional Chinese medicine for 3,500 years, and different forms of mercury are still used in all aspects of life. For example, Cinnabar is highly toxic because it contains mercury. However, the toxicity can be removed after grinding and washing (called Shui-Fei). Hence, it has become a good medicine for tranquilizing and relieving convulsion (Jain et al., 2019). Inorganic mercury, such as ethylmercury and aminosalicylic acid, is used in vaccines, pharmaceuticals, and cosmetics. External preparations are indispensable and unique treatment measures in traditional Chinese medicine (TCM) surgery. Among them, external preparations containing mercury have the characteristics of removing slough and promoting the growth of tissue regeneration, sterilization, anti-inflammation, detumescence, and relieving pain, as well as promoting wound repair. Zuotai, another preparation containing mercury, is one of the most famous and important medicinal materials in Tibetan medicine. Zuotai can improve the curative effect, reduce the toxicity of other drugs, and has significant effects in anti-convulsion, anti-inflammation, antipyresis, and enhancing immunity. Menggen Wusu, a commonly used mercury-containing mineral preparation in Mongolian medicine, is mainly used for the treatment of gout, rheumatism, scabies, etc. (Zhou et al., 2017).

However, in today’s world, mercury is considered as a harmful environmental pollutant and one of the most dangerous toxins. As mineral drugs mostly contain arsenic, mercury, and heavy metals, they should be avoided in new drug research, so the use of minerals in drugs is becoming less and less. Because people were concerned for the environment and human health, the large numbers of applications of mercury in production processes such as industry and agriculture have aggravated environmental mercury pollution, and mercury poisoning has become a concern. Most elements have multiple valence states and forms in nature, and different forms often show different properties and toxicity. European Union legislation requires the detection of mercury, selenium, arsenic, and tin not only by measuring the total content but also by distinguishing between different forms of content. Existing Chinese patent medicines such as Angong Niuhuang Pill, Niuhuang Jiedu Pill, and so on have been restricted to use. To meet the export requirements, mineral drugs such as realgar and cinnabar have also been canceled. The application scope of mineral drugs has a trend of further narrowing. In recent years, the research on Nano-Chinese medicine has become increasingly hot. The Ministry of Science and Technology of the People’s Republic of China and other countries have funded the research on Nano-realgar, sulfur, and calamine. Nano-drugs can improve bioavailability and reduce toxicity, opening up a new prospect for the application of mineral drugs. In the environment, mercury is mainly composed of elemental mercury, inorganic mercury, and organic mercury (the combination of bivalent mercury and alkyl). Inorganic mercury is synthesized into organic mercury through biological activity. The toxicity of mercury to the human body depends to a large extent on its existing form. Mercury poisoning is the strong combination of -S-H- and -S-S- bonds on enzyme protein *in vivo* in the human body, which denatures protein and inactivates the enzyme, thus causing structural and functional damage to tissue cells (Yang et al., 2020).

Human mercury exposure is primarily through inhalation of elemental mercury vapor through occupational or dental amalgam exposure or ingestion of mercury combined with organic moieties (methyl, dimethyl, or ethyl mercury), primarily from marine products (Babic, 2014). According to the World Health Organization (Ajiboye et al., 2020), most human exposure to metallic mercury results from the release of mercury vapor from amalgam fillings at a rate of 2–28 μg per day per side, with about 80 percent of this absorbed (Cariccio et al., 2019). A less common source of mercury vapor is spilled mercury, severe acute exposure to mercury vapor due to vacuum treatment of spilled mercury, eventually causing idiopathic thrombocytopenic purpura in humans (Cao et al., 2015).

Methylmercury and dimethylmercury (organomercury) are usually derived from biological sources, mainly freshwater or saltwater fish. The only sources of human exposure to methylmercury are food fish and marine mammals (Schwartz et al., 1992). Methylmercury is produced in the environment by biomethylation of inorganic mercury in aquatic sediments. As a result of mercury pollution, more than 3,000 lakes in the United States have been closed to fishing and many marine fishes are also contaminated with relatively high concentrations of mercury (Genchi et al., 2020). Cases of severe and even fatal methyl mercury poisoning date back to England in the 1860s, accidents in Iraq in 1971 and 1972 killed hundreds of people and caused thousands of serious poisoning incidents and industrial discharges of methyl mercury in Minamata bay and the Agana River in Japan resulted in the accumulation of toxic substances in fish (Jackson, 2018). In the Minamata Bay area, infants are beginning to develop serious illnesses similar to cerebral palsy, and fetuses are considered more sensitive to methylmercury than adults (Iwata et al., 2016).

Although mercury is the second most common cause of heavy metal poisoning, chronic mercury poisoning is more common and mainly occurs in production activities, and the clinical phenomenon of mercury poisoning has received little attention. In 1997, ATSC reported a total of 3,596 mercury exposures nationwide. Mercury and its derivatives have been used as anti-parasitic, anti-syphilis, antipruritic, preservative, anti-inflammatory, diuretic, dental amalgam, and substitute for over 3,000 years. Although the industrial use of mercury is increasing, occupational mercury exposure is currently recognized in more than 60 industries, including the manufacture of glass thermometers, batteries, barometers, neon lights, paper, paint, jewelry, pesticides, fungicides, chlorine and caustic soda, and in dental practice (Kosnett, 2013). From it is the belief that the use of mercury in medicine is problematic to advocacy for more substantial scientific testing of controversial drugs rather than outright rejection, mercury and mercury-containing preparations have been time-tested traditional substances that are indispensable to medicine. Contemporary proponents of mercury as a drug argue that mercury-containing formulations are not only safe for human use but also the most effective treatment if their traditional procedures for purifying and detoxifying mercury are strictly followed. Researchers studied the toxicity of mercury and its poisoning mechanism through animal experiments (Jackson, 2018). However, to confirm its toxic effect on humans, a clinical toxicity study of mercury is also essential. A total of 3,596 mercury exposures were reported nationwide. For more than 3,000 years, mercury and its derivatives have been used as anti-parasitic drugs, anti-syphilis, antipruritic, antiseptics, anti-inflammatory drugs, diuretics, dental amalgams, and substitutes. Although the industrial applications of mercury are increasing, there are currently recognized occupational exposures to mercury in more than 60 industries, including the manufacture of glass thermometers, batteries, barometers, neon lights, paper, paint, jewelry, pesticides, fungicides, chlorine, and caustic soda. From arguing that the use of mercury in medicine is problematic, to arguing for more substantive scientific testing of controversial drugs rather than outright rejection, furthermore, impassioned calls to defend mercury and its formulations as time-tested substances indispensable to medical tradition. Contemporary scholars who support mercury as a medicine believe that if the traditional procedures of purifying and detoxifying mercury are strictly followed, mercury-containing preparations are not only safe for human use, but also the most effective treatment method. Researchers have studied the toxicity of mercury and its poisoning mechanism through animal experiments, but to confirm its toxic effects on humans, clinical toxicity studies of mercury are also indispensable (Clarkson et al., 2003).

In response to the current controversy over the use of mercury, this article reviews the use history, clinical application, pharmacology, and toxicology of mercury and mercury-containing preparations. The article could be served as a reference for traditional medicine practice from all over the world to scientifically understand the value and toxicity of mercury.

### Application History of Mercury

#### Application History of Mercury in China

Mercury is commonly known as *Shuiyin* in China. The distribution in nature is extremely small, and it is considered a rare metal, but mercury has been discovered very early. Natural mercuric sulfide, also known as Cinnabar, because of its bright red color, has long been used as a red pigment. According to the Yin ruins (1319 B.C.-1046 B.C.) unearthed in Oracle Bone Inscriptions coated with Cinnabar, can prove that China used natural mercury sulfide before history. According to the ancient Chinese literature, before the death of Qin Shihuang (259 B.C.-210 B.C.) (Liu, 2008), some princes had already used mercury in their tombs. For example, Qi Huangong (685 B.C-643 B.C.) was buried in Linzi County, present-day Shandong Province, with a pool of mercury in his tomb. That is to say, China has been able to obtain large amounts of mercury in the 7th century B.C. or earlier. At that time, it was known to use Cinnabar to smelt mercury, and initially, it was a crude low-temperature roasting method. During the Wei, Jin, and Southern and Northern Dynasties (220 B.C. -589 B.C.), people held high the Taoist theory of immortality. In Taoist thought there is a kind of theory, that is, to use the external things to consolidate the human body, and botanical drugs even can’t deposit for a long time, so can’t be used to longevity. Taoism mainly uses metal and stone as the medicine of immortality, which happens to coincide with early alchemy. Taoist external alchemy came into being in this environment. Substances such as Cinnabar, mercury, gold, and lead played an important role in external alchemy. Dansha was changed into mercury (Su, 2008). The ancient Chinese people put the Cinnabar (HgS) in the air and calcined it to obtain mercury. However, the generated mercury is easily volatilized and difficult to collect, and mercury poisoning may occur to operators. The Chinese people have accumulated experience in practice and used closed methods to make mercury. Some are closed in bamboo tubes, and some are closed in pomegranate-shaped ceramic pots (Maqbool et al., 2017).

In ancient China, mercury was used as a surgical drug in traditional Chinese medicine. Mercury compounds, which had the effects of disinfection, laxation, and diuresis, were no longer used or rarely used. *Fifty-two Prescriptions* (before 168 B.C.) in silk manuscripts unearthed in Changsha’s Mawangdui Han Tomb in 1973 was copied in the Qin and Han Dynasties (221 B.C.-206 B.C.), which recorded the oldest Chinese medical prescription in the excavated literature, probably in the Warring States Period (403 B.C.-221 B.C.) Mercury is used in four of these compounds, for example in the treatment of scabies with a mixture of mercury and realgar. It is recorded in the Classic of *Shen Nong Ben Cao Jing* (A.D. 25–A.D. 220) that Dansha is slightly cold and can govern the body pain and various illnesses of the five Zang-viscera, nourish the spirit, soothe the soul, replenish *Qi*, improve eyesight, kill the essence and the evil spirits, and soothe the gods for a long time before aging. Dansha is non-toxic, and it regulates blood vessels, relieves vexation and thirst, benefits spirit, and delights people (Tao, 1986). It can eliminate medium and evil, abdominal pain, toxic gas, scabies and phlegm, and various sores, and can be used to subdue immortals. It is stated in *Ben Cao Gang Mu* (A.D. 1518-A.D. 1593) that red sand can cure carbuncle caused by fright, remove fetal toxin, pox toxin, drive away pathogenic factors, and induce sweating. Thus, Cinnabar (HgS) has been used for medicinal purposes for more than 1000 years in China, mainly for clearing heart fire and relieving convulsion, tranquilizing the mind, improving eyesight, and detoxicating.

The toxicity of mercury was recorded in *Ben Jing Feng Yuan* (A.D. 1617-A.D. 1700) in the Original Classic of Nature: Mercury is very poisonous and cannot be oral. One day, a person accidentally ate mercury and fell heavily in his abdomen. For curing, he used 2 kg of pork fat, cut into small pieces and roasted, and mixed with raw honey to eat.

#### Application History of Mercury in India

Ayurveda, Unani, and Siddha in India have a history of mercury use. In Ayurveda, mercury is known as Rasa because it can slow the aging process, cure many diseases and delay death. Mercury is mainly used to treat syphilis. In the 16th century, a new disease appeared in India. It was first called phiraṅgaroga by Ayurvedic classics Bhāvaprakāśa and it was classified as Āgantu by Bhavamisr, which translates as exogenous or invasive because this disease entity enters the body from the outside, since the disease entity comes from the outside of the human body, the humoral complications are secondary development and must be identified by its symptoms. He divided phiraṅgaroga into three, or perhaps three, stages: external, internal, external, and internal. Bhāvamiśra (Dole and Paranjpe, 2004) describes seven different treatments, five with mercury in their prescriptions, three with mercury in their prescriptions, one with mercury as a fumigant, and one with mercury rubbed into the hands of patients. Mercury is unique for the treatment of syphilis. The main prescription for the treatment of phiraṅgaroga by Bhāvamiśra describes the ingestion of rasakarpūra (mercury like camphor, white powder), which is either mercurous chloride (calomel), or mercuric chloride (corrosive sublimate), or a mixture of the two (Dagmar, 2015).

Mercury is still very popular in Unani medicine and mercury preparations are often used for fumigation or pills. Mercury is very useful for treating illnesses caused by large amounts of cold and dampness, and older (Persian) works emphasize the heating and astringent therapeutic effects of mercury. Mercury is said to be very useful in the treatment of ulcers, especially those associated with syphilis. It is also used for treating hemiplegia, facial paralysis, or spasm. All the information on the use of mercury in drugs of Unani emphasizes the importance of purified mercury. Ḥakīm Ajmal Khān wrote that ḥakīms must use purified mercury. He mentioned a total of eight methods of purifying mercury, some of which he believed were used by ayurvedic yogis, and some of which were specifically used by yogis. The purification process removes the undesirable parts and toxic effects of the drug. This increases the intensity of a specific drug while improving its efficacy and potency. Ḥakīm Kabīrud Dīn and point to several advantages of kushtajāt: small amounts work, and they are easier to take than other medications. Several formulations containing Marham-e shingraf were recommended to use external mercury preparations (Dagmar, 2015). Because it is thought to be hot and dry. Shingraf is also thought to be very effective against kidney ulcers (qurihe gurdeh) and other malignant ulcers, helps relieve pain from dry skin and syphilis, and is a very potent aphrodisiac. It is a local custom in India to use shingraf as an anticonvulsant and to treat skin diseases. It was commonly used as a fumigant. In this case, people throw Cinnabar on the red-hot iron plate, and the patient covers himself with a blanket and sucks in the hot air from the censer. Kushta shingraf is also considered to be a very powerful drug for sexual decline or anemia. It can treat all diseases, also known as cold dampness, which is caused by excessive phlegm. This includes a general weakness or loss of appetite (Dole and Paranjpe, 2004).

#### Application History of Mercury in Europe

In the western chemical history data, which has been recorded in the Egyptian tomb found a small tube of mercury; according to textual research is the product of the 16th-15th century B.C., perhaps as a preservative. Western alchemists believed that mercury was the common denominator of all metals-the embodiment of metallicity. What they think of as metallic is an element that makes up all metals. Compounds of mercury were also used on the skin. Some of the earliest uses being as pigments in red ink were used by alchemists. The Spanish galleon sent 76 pounds of liquid mercury to the Americas to extract gold and silver. In the middle Ages, mercury was widely used by alchemists because it was able to form amalgam and attempted unsuccessfully to convert mercury into gold. During this period, two mercury compounds, mercurous chloride (calomel) and mercuric chloride (corrosive sublimate) were produced for the first time (Maqbool et al., 2017).

Mercury and mercury compounds were increasingly used in medicine beginning in the 16th century AD, especially after their use was advocated by Paracelsus (1493–1541). At the same time, with the colonial expansion in Europe, trade-in mercury became a global phenomenon, especially for gold and silver mining and medical purposes. Over the next four centuries, mercury compounds such as corrosive sublimation (HgCl), calomel (Hg_2_Cl). Mercury sulfide compounds (HgS) were the most important and commonly used drugs in European and Asian pharmacology, such as the treatment of inflammation of the nose and throat, corneal stains, ulcers, and warts, etc. As a laxative, it can stimulate the function of the biliary tract, and prevent diarrhea, vomiting, and treat edema. The treatment of spleen, liver, lung diseases, most notably, can prevent syphilis (Clarkson, 2002). Paracelsus is a passionate advocate. He has an immortal motto; Dose produces the toxin, syphilis treatment sometimes lead to severe poisoning.

In the 20th century, mercury salts were long used in felt hats because they were found to be very effective in making animal hair sticky, resulting in high-quality felt. Organic mercury, especially methylmercury and ethylmercury, is widely used in agriculture as an antifungal agent in seed grains, but this practice has been discontinued due to the high incidence of poisoning in humans and certain wildlife species. As a preservative, mercurochrome (dibromo-hydroxymercury fluorescein) has been widely used and has been the cause of several documented poisoning incidents (Kopec et al., 2018). Thiomersal (ethylmercury thiosalicylate) is still used in vaccines worldwide, although the United States is currently concerned about its potential toxicity to infants. The use of phenyl and ethyl mercury compounds as antiseptic antibacterial agents is still limited. It is also used as a spermicide in chemical contraceptives and antiseptics, and it is still used in dental amalgam, vaccines (such as the organic mercury compound thimerosal), cosmetics, eye drops, and saline solutions. Its uses in the industrial age include mercury barometers and thermometers, as electrodes for the production of chlorine and caustic soda from brine electrolysis (Rocha et al., 2012), and in electronic switches that are still widely used today. Mercury is also widely used in mercury arc lamps and incandescent lamps.

In general, most health authorities are careful to control human exposure to various forms of mercury. However, with the outsourcing of manufacturing and other activities previously located in developed countries, the risk of mercury contamination of imported materials now persists.

### The Clinical Application of Mercury and Mercury-Containing Preparations

**FIGURE 1:**
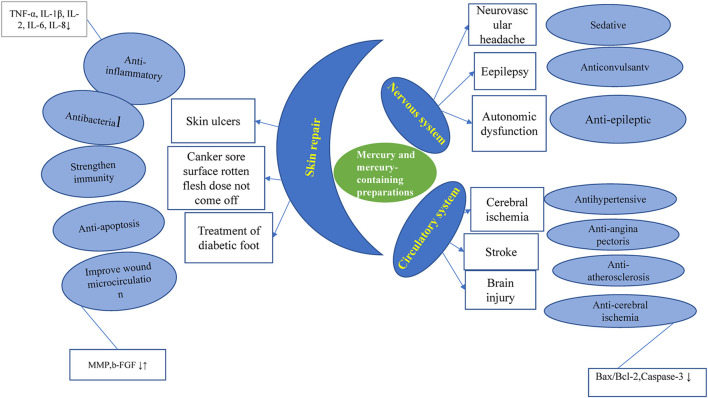
Diseases and pharmacological effects of mercury and mercury-containing preparations.

The compositions and clinical applications of mercury-containing preparations commonly used in traditional Chinese medicine and minority medicine are summarized in Table 1. Figure 1 is the summary of the clinical application and pharmacology of mercury preparations.

**TABLE 1:** Clinical application of mercury and mercury-containing preparations in traditional Chinese medicine.

Common name and the form	Chemical composition containing mercury	Category	Included in the Chinese pharmacopoeia (2020 Edition)	Clinical application	References
Cinnabar	HgS	Chinese medicinal materials	Yes	Palpitation, insomnia, dreaminess, epilepsy, infantile convulsion, blurred vision, aphtha, sore throat, sore and swollen poison, etc.	Jain et al. (2019); Li et al. (2016b); Ding (2012); Zhu et al. (2014); Guan (2002); Yang et al. (2012)
Baijiang Dan (Pill)	HgCl_2,_Hg_2_Cl_2_	Chinese medicinal materials	No	It mainly plays a role of corrosion, so that the sore tissue can be corroded and withered	Su et al. (1991); Zhao (1983); Zhou (1982)
Hongsheng Dan (Pill)	HgO	Chinese medicine preparations	No	Furuncle carbuncle, Fistula sinus; Goiter scrofula; Breast cancer and carbuncle; Scabies; Eczema; Syphilis; All stubborn sores long collapse not folding; Dark purple black; Pus discharge is not smooth; Carrion does not go; The new tissue grows slowly	Shao (2016); Chen (2013); Liu (2008); Wei et al. (1990); Li et al. (2013b)
Angong Niuhuang pills (Pill)	HgS	Chinese medicine preparations	Yes	Cerebrovascular diseases, meningitis, toxic encephalopathy, sepsis and fever coma caused by a variety of reasons	Hu and Ma (2015); Li et al. (2013a); Lu et al. (2010); Liu et al. (2017); Yin (2011); Ni et al. (2012); Zhu (2007)
Shengji Yuhong Gao (Paste)	Hg_2_Cl_2_	Chinese medicine preparations	No	Rotten tissue difficult to take off, new tissue is not raw, or rot has taken off, new tissue is not raw, long not convergent sore surface	Zhang et al. (2019a); Xu et al. (2021); Dong and Liu (2015); Feng et al. (2008); Li and Zhang (2010)
Zhuhong Gao (Paste)	HgS, HgO	Chinese medicine preparations	No	No slough or granulation growth on the sore surface of the ulcer, or high protrusion of the sore mouth, or accompanied by red swelling and hot pain around the sore, yellow and thick pus, or dark sore surface, rare pus, etc.	Wang et al. (2016); Yan et al. (2013); Mao and Li (2003); Wang et al. (2013)
Huafeng Dan (Pill)	HgS	Chinese medicine preparations	No	Wind phlegm obstruction (headache, tinnitus, cerebral arteriosclerosis, limb numbness and weakness, and brain atrophy), stroke hemiplegia, epilepsy, facial paralysis, facial paralysis, facial distortion, etc.	Chen et al. (2020); Chen (2013); Yan (2017)
Menggen Wusu Shibawei pills (Pill)	HgS	Mongolian medicine preparation	No	Gout, bakanae disease, rheumatism, yellow water disease, scabies, etc.	Shi et al. (2015); Bai et al. (2020); Wang et al. (2020)
Zuotai	HgS	Tibetan medicinal materials	No	Apoplexy, paralysis, hypertension, nerve disorder, cardiovascular disease, hepatobiliary disease, impotence, gastrointestinal disease, tumor, etc.	Zhu et al. (2013); Jiang et al. (2009); Ceng and He (2005); Huang et al. (2013)
Qishiwei Zhenzhu pills (Pill)	HgS	Tibetan medicine preparation	Yes	Ischemic stroke (cerebral ischemia, stroke, paralysis, sequelae of stroke, lacunar infarction, cerebral hemorrhage), cerebral concussion, hypertension, infantile convulsion, facial paralysis, Alzheimer’s disease, neurovascular headache, autonomic nerve dysfunction and angina pectoris and other cardiovascular, cerebrovascular and nervous system diseases	Hu and Bai (2012); Fu et al. (2021); Jiang (2010); Liang et al. (2019); Hai (2002); Xu et al. (2020); Xu et al. (2019); Wang and Zhou (2001); Chen (1999)

#### Cinnabar

Cinnabar, also known as Dansha and Censha in Chinese, is a sulfide Cinnabar, which is mainly composed of mercury sulfide (HgS) and also contains a small amount of soluble mercury (Yang et al., 2012). It has the effects of clearing heart fire, relieving convulsion, tranquilizing the mind, and detoxicating. It is mainly used for the treatment of palpitation, insomnia and dreaminess, epilepsy, infantile convulsion, blurred vision, aphtha, sore throat, sore and swollen poison. It is a commonly used medicinal material in TCM clinical practice. Among the 564 preparations recorded in Pharmacopoeia of the People’s Republic of China (2005 edition), the preparation containing Cinnabar accounted for about 7.6% of the total. HgS is highly insoluble in aqueous solution and is difficult to be absorbed by the human body. It can only be dissolved in aqua regia or alkaline sulfide solution to form complex anions and composite sulfide. However, Cinnabar still has strong toxic and side effects because it often contains a small number of soluble mercury salts such as HgCl.

#### Baijiang Dan (Pill)

The chemical composition of Baijiang Dan is mercuric chloride (HgCl_2_), which has a strong corrosive effect. It is toxic and should not be taken orally. When in use, it is ground into powder and sprinkled on the sore head, mixed with other medicines to be ground into powder and mixed for application, and bound up with a plaster cover dressing, or used as a dressing. It mainly plays a role of corrosion, so that the tissues with abnormal sores can be corroded and withered (Zhang and Pu, 2019).

#### Hongsheng Dan (Pill)

Hongsheng Dan, also known as a red powder, is red oxidized mercury refined from mercury, sal Nitri, alumen, Cinnabar, realgar, and melanteritum which have the functions of removing toxin and pus, removing slough and promoting the growth of tissue, killing parasites and drying dampness (Liu and Zhang, 2008). It is mainly used for treating furuncle and carbuncle, fistula and sinus tract, goiter and scrofula, breast cancer and carbuncle, scabies, eczema, syphilis, all persistent sores, dark purple and black, unsmooth purulence, rotten meat, and raw new meat. Hongsheng Dan is corrosive and toxic. It is used in combination with other medicinal materials in clinical application, for example, Hongsheng Dan is combined with plaster of Paris in a ratio of 1: 9, 2: 8, and 1: 1 to prepare *Jiu Yi Dan*, *Ba Er Dan*, and *Wu Wu Dan*, etc. The higher the content of Hongsheng Dan (Shao, 2016), the stronger the effect of pus extraction and corrosion removal. Studies have shown that mercury ions can be combined with the sulfhydryl group of bacterial enzymes to inactivate the enzymes and lead to bacterial death. The red powder has a good antibacterial ability against experimental strains and a strong bactericidal effect against common suppurative bacteria, such as *Staphylococcus aureus* and *Escherichia coli*. The bactericidal effect of Hongsheng Dan is more than 100 times that of carbolic acid. The mercury ion in Hongsheng Dan can make the pathological tissue protein in contact with drugs coagulate and necrotic, and gradually separate from the healthy tissue to remove the rot. At the cellular level, its mechanism may be related to increasing the inflammatory response of wound granulation and promoting the infiltration of inflammatory cells and the shedding of necrotic tissue in the wound (Liu, 2008).

#### Angong Niuhuang Pills (Pill)

Angong Niuhuang pills, from Wu Jutong’s Wen Bing Tiao Bian, Vol. 1, Shangjiao (1798), is composed of *Bos taurus domesticus* Gmelin, *Stapelia luxurians* Dammann ex Rust., Pernulo, Moschus, Cinnabar, Realgar, *Coptis chinensis* Franch., *Gardenia jasminoides* Ellis., *Scutellaria baicalensis* Georgi., Borneol. It has a significant curative effect on coma, delirium, convulsion, high fever, and irritability. Angong Niuhuang pills, an emergency drug in traditional Chinese medicine (TCM), is widely used for cerebrovascular diseases, meningitis, toxic encephalopathy, sepsis, and various diseases caused by fever, which is included in Pharmacopoeia of the People’s Republic of China (2020 edition). Cinnabar (10%) and Realgar (10%) are the main components of Angong Niuhuang pills. Angong Niuhuang pills are clinically used for more acute hemorrhagic stroke than for acute ischemic stroke and its effect is better than that of ischemic stroke. Angong Niuhuang pills can significantly improve the consciousness disorder of elderly patients with severe cerebrovascular diseases and help restore the neurological dysfunction of patients (Hu and Ma, 2015).

At present, Xingnaojing Injection prepared from Angong Niuhuang pills has been reported clinically to be capable of reducing blood viscosity and improving brain circulation. It is especially suitable for patients with acute cerebral infarction with symptoms of coma, hemiplegia, mouth, and tongue deviation, strong tongue and unsmooth language, limb numbness, etc (Li Q. B. et al., 2013).

#### Shengji Yuhong Gao (Paste)

Shengji Yuhong Gao is one of the most commonly used external drugs in surgery of traditional Chinese medicine. Its main medicinal materials include *Glycyrrhiza uralensis* Fisch., *Angelica dahurica* Benth., *Angelica sinensis* Diels., *Lithospermum erythrorhizon* Sieb., *Fraxinus chinensis* Roxb., *Daemonorops draco* Bl., light powder (Hg_2_Cl_2_) (Liu and Jiang, 2010). It has the effect of removing slough and promoting the growth of tissue. It is mostly used for those who have difficulty in getting rid of rotten tissue and those whose new tissue is not raw, or for those whose sore surface has been stripped of rotten tissue and whose new tissue is not raw and has not been closed for a long time. In addition to being used for various chronic ulcers. It is also used for the treatment of a multicenter, randomized, double-blind, and controlled clinical trial study of the Gao in the treatment of chronic ulcers of the lower limbs showed that after 2 weeks of intervention. The exudation of the wound surface, the growth of granulation tissues, the shedding of necrotic tissues, the bacterial culture of exudates, and other aspects were significantly improved, and the optimal effect was achieved within 4 weeks. The total effective rate was 99.21%, and the wound surface area was reduced by 81.6%, preliminarily indicating the relationship between the administration time and the curative effect (Cai et al., 2007). The total effective rate was significantly higher than that of Vaseline and was consistent with the result of Shengji Yuhong Gao in the treatment of leprosy ulcers on the sole. Shengji Yuhong Gao can promote wound healing by increasing the levels of hemoglobin and hydroxyproline in wound granulation and also improving the granulation color, ulcer depth, and granulation coverage.

#### Zhuhong Gao (Paste)

Zhuhong Gao consists of Cinnabar (containing HgS and soluble mercury salt) and red powder (containing HgO and soluble mercury salt) and has the effect of removing slough and promoting the growth of tissue. It is commonly used in clinics in the form of gauze (Wang L. et al., 2012). It contains 1.8 mg Gao per square centimeter. After 4 weeks of treatment of chronic ulcers, the total effective rate is 84.2%. At the early stage of drug administration, the dose of the crude drug Zhuhong Gao was greater than 13.6 mg/cm^−2^, which played a role in removing slough. When the pus rot was removed, the corrosion of new granulation tissue appeared after continuous application, resulting in slow wound healing and decreased efficacy. The dosage of crude drug 1.7–6.8 mg/cm^−2^ was effective for promoting pus and removing putrefaction, and the dosage groups 1.7 and 3.4 mg/cm^−2^ showed good effects of removing slough and promoting the growth of tissue regeneration. The granulation tissue on the sore surface was fresh, and it healed quickly. In the early stage of administration, the dose of Zhuhong Gao was greater than 13.6 mg/cm^−2^, which preliminarily revealed the relationship between dose, stage of sore and curative effect (Wang et al., 2013).

#### Huafeng Dan (Pill)

Huafeng Dan is a traditional Chinese patent medicine prepared from 15 Chinese medicinal materials including *Gastrodia elata* Bl., *Bombyx mori* Linnaeus, scorpion, *Gastrodia elata* Bl., *Nepeta cataria* L., Realgar, *Leonurus japonicus* Houtt, Moschus, Cinnabar. Chinese patent medicine is made by fermentation (Chen et al., 2020). It is used for the treatment of wind phlegm obstruction (headache, tinnitus, cerebral arteriosclerosis, limb numbness and weakness, and cerebral atrophy), stroke hemiplegia, epilepsy, facial paralysis, and facial paralysis. In 1951, it was listed as one of the four famous Chinese medicines under the protection of the State Council. The original prescription of Wansheng Huafeng Dan contains about 10% realgar and Cinnabar. It is well known that realgar and Cinnabar are compounds of arsenic and mercury, respectively. Li et al. (2013a) selected 60 patients with acute cerebral infarction. The results of the study showed that Huafeng Dan had a significant intervention effect on Hcy, ET, and NO of acute cerebral infarction, and confirmed that the curative effect of Huafeng Dan combined with western medicine was superior to the simple western medicine treatment group. The curative mechanism might be through reducing the high-risk factors of stroke, Hcy, and ET, increasing NO and reversing the vascular endothelial function, and thus promoting the recovery of neurological function. Chen (2013) carried out a clinical effectiveness trial of Huafeng Dan in the treatment of stroke. Sixty of stroke patients were selected from Chen Yong brain. After treatment, the Fugl Meyer motor function scale (FMA) and activity of daily living (ADL) scores of the Huafeng Dan experimental group were superior to those of the conventional western medicine clopidogrel sulfate control group, indicating that the application of Huafeng Dan had significant efficacy in the treatment of stroke with less adverse reactions, which could provide a certain reference for the clinical treatment of stroke.

#### Menggen Wusu Shibawei Pills (Pill)

Menggen Wusu as Mongolian medicine clinical commonly used mineral medicinal materials is very commonly used in Mongolian medicine preparation. Menggen Wusu main components for HgS and elemental sulfur, pungent, cold, heavy, toxic, its processed products with dry *Xie Ri Wusu*, dry purulent blood, insecticidal, the efficacy of carbuncle (Chun and Battelle, 2013). The proportion of Menggen Wusu in different Mongolian medicine preparations also varies greatly. Among them, Menggen Wusu in Menggen Wusu Shibawei pills is high in content, widely used in clinical practice and has a significant curative effect. Menggen Wusu Shibawei pills were written in the classic Mongolian medical book *Guan Zhe Zhi Xi* (1888). In its prescription, the mercury-making main drug with the main effects of drying *Xie ri Wusu* and killing Mu was used, together with other medicinal materials such as Mercury, Wenguan Mugao, Sulfur, *Abutilon theophrastii* Medic., *Aucklandia lappa* Decne., *Myristica fragrans* Houtt., *Nardostachys jatamansi* DC., Resina Liquidambaris, *Terminalia chebula* Retz., *Aconitum kusnezoffii* Reichb., *Amomum tsao-ko* Crevost., Acorus tatarinowii, Semen Cassiae, Suge Mule, *Carthamus tinctorius* L., Gypsum. Menggen Wusu Shibawei pills have a wide range of clinical applications. The majority of the conditions it treats are sticky (A theoretical term of Mongolian Medicine), dry yellow water (A theoretical term of Mongolian Medicine) and various diseases related to it, including dermatology, rheumatology, respiratory medicine, digestive medicine, and gynecology and so on. Among them, dermatology is the most commonly used disease, accounting for 76.23%. The specific diseases include psoriasis, eczema, urticarial, scabies, and pruritus. The best ones have reached 100%, and the course of treatment lasts for up to 3 months. The total effective rate for treating the diseases of the rheumatology immunology department is up to 97.5%. Treatment of respiratory diseases accounted for 6.4%, the total effective rate of 100%. The rate of treatment for digestive diseases was 1.3%, and the effective rate was 93.3%. Saren Garidi is used for the treatment of rheumatic arthritis with the main medicinal material being Menggen Wusu, which dries “yellow water” (A theoretical term of Mongolian Medicine) and “kills Chong” (A theoretical term of Mongolian Medicine) (Chun and Battelle, 2013).

#### Zuotai

Known as *King of Nectar’s Essence*, Zuotai is one of the most famous and important medicinal materials in Tibetan medicine (Zhu et al., 2014). It is a black and blue powder with an unparalleled curative effect. It has certain pharmacological activities of calming the nerves, promoting sleep, anticonvulsant, antipyretic, anti-inflammatory, enhancing immunity, prolonging the life of female fruit flies, and bacteriostatic. Natural medicinal materials such as liquid mercury (mercury), sulfur, energy pool eight metal, energy pool eight ore, are processed by senior Tibetan medical practitioners using special processing technology. The general preparation process is that mercury must be decocted with botanical and animal medicine, calcined, and processed into ashes to retain the efficacy and remove toxic side effects (Li W. Z. et al., 2016). Zuotai is generally not used alone but is usually used for assisting medicinal materials to form a compound with other medicinal materials. Tibetan doctors have always believed that Zuotai can improve the curative effect and reduce the toxicity of other drugs. The compound preparation containing Zuotai is widely used in the clinic. Compound preparations containing Zuotai are widely used in clinical practice, such as the use of Tibetan medicine preparation Dayuejing Pills in the treatment of peptic ulcers. As early as 1300 years ago, Zuotai has been used as a key component of the *Renqing Series* Tibetan medicine. For example, *Renqing Manjue* (pills) is clinically used to treat arrhythmia and middle retinal artery embolism, and *Renqing Changjue* (pills) is used for treating digestive system diseases such as liver cirrhosis, chronic hepatitis, and peptic ulcer. Up to now, it is still used as one of many preparations, mainly for the treatment of stroke, paralysis, hypertension, neurological disorders, cardiovascular diseases, hepatobiliary diseases, impotence, gastrointestinal diseases, tumors, etc (Huang et al., 2013).

#### Qishiwei Zhenzhu Pills (Pill)

Qishiwei Zhenzhu pills are mainly composed of Zuotai (medicinal materials containing mercury), Pearl, Niuhuang, and other 70 rare Tibetan medicinal materials. It is a black pill processed by traditional Tibetan medicine processing technology combined with modern pharmaceutical means and has been included in *Pharmacopoeia of the People’s Republic of China* (2020 edition).

Qishiwei Zhenzhu pills have the effects of tranquilizing, tranquilizing, dredging channels, activating collaterals, harmonizing *Qi* and blood, resuscitation, and opening orifice (Hu and Bai, 2012). Continuous practice of generations of Tibetan physicians and clinical observation in recent years have shown that the drug can treat black and white pulse disease and dragon blood disorder, stroke, paralysis, hemiplegia, epilepsy, infantile convulsions, cerebral concussion, cerebral hemorrhage, postoperative recovery from brain diseases, heart disease, hypertension, autonomic nerve dysfunction, and other nervous system diseases. In the clinic, the Qishiwei Zhenzhu pills have remarkable curative effects on many diseases, such as ischemic stroke (cerebral ischemia, stroke, paralysis, SARS, and sequelae of stroke), lacunar infarction, cerebral hemorrhage concussion, hypertensive infantile convulsion, facial paralysis, Alzheimer’s disease, neurovascular headache, autonomic nerve dysfunction, angina pectoris, and other cardiovascular, cerebrovascular and nervous system diseases, which is equivalent to the category of black and white pulse disease in Tibetan medicine. Judging from the clinical application range of the Qishiwei Zhenzhu pills, the treatment of cardiovascular and cerebrovascular diseases and nervous system diseases is expanded to other diseases, such as infantile convulsion, rheumatic arthritis. The combination of Qishiwei Zhenzhu pills with other Chinese and western medicines achieved satisfactory results and no adverse reactions were reported. For example, the total effective rate of the combination of treatment for lacunar infarction with Ginkgo biloba injection was 90.6%, and the total effective rate of the combination of treatment for cerebral infarction with Twenty-five taste Pearl pills was 96.7% (Hu and Bai, 2012).

### Pharmacology of Mercury and Mercury-Containing Preparations

#### Cinnabar

Cinnabar can inhibit the excited central nervous system, playing sedative and hypnotic effects. External use can inhibit and kill skin bacteria and parasites. Rabbits were intragastrically administered 0.1–0.2 g/kg Cinnabar, which increased the amount of total nitrogen excreted from rabbit urine (Guan, 2002). Cinnabar can soothe the nerves, inhibit convulsion. Zhu and Sun (2014) explored that the medium and high doses of Cinnabar Anshen Pill water decoction could significantly reduce the awakening time of insomnia rats and prolong the total sleep time of insomnia rats, and significantly improve the sleep of insomnia rats. In the anti-arrhythmia aspect, Li Q. et al. (2016) discovered in their research that the anti-arrhythmia effect and tranquilization effect of Zhusha Anshen Pill were much stronger than that of Zhusha Anshen Pill which don’t contain Cinnabar, confirming the status of Cinnabar as the sovereign drug in the prescription. Zhu (2007) studied the effect and mechanism of Cinnabar and realgar in Angong Niuhuang pills against brain injury in rats, and determined the protective effect of Cinnabar on brain injury. It also be explored the antiviral pharmacological effects of Cinnabar and realgar, and illustrated the scientific principle of inactivation of viral protease by Cinnabar (mercuric sulfide) as a heavy metal. Diseases clinically treated using mercury and preparations are summarized in (Table 1).

#### Baijiang Dan (Pill)

Baijiang Dan, with a long history of use and distinguished therapeutic effect, plays an important role in the scope of external medicine for surgery in traditional Chinese medicine. Baijiang Dan has the functions of detumescence, removing toxins, removing rot, killing parasites, and so on. It is used in the early, middle, and late stages of curing toxic carbuncle and tumors. It has a good effect on skin diseases, gynecological diseases, hemorrhoids, rheumatism, and all kinds of pain. The research by Su et al. (1991) showed that the minimum inhibitory concentration of Baijiang Dan on six different bacteria was completely equal to its minimum bactericidal concentration, which might be related to its pharmacological effect that mercury ion combined with HS of bacterial respiratory enzyme lost its activity and died due to breathing. The inhibitory concentrations of Baijiang Dan against *Staphylococcus aureus* and B hemolytic streptococcus in Gram-positive bacteria were 0.08 μg/ml and 3.90 μg/ml, respectively, and against *Escherichia coli* and *Pseudomonas aeruginosa* in Gram-negative bacteria were 0.98 μg/ml and 0.63 μg/ml, respectively. Baijiang Dan also had a significant killing effect on drug-resistant *B. subtilis*. Its concentration was the same as that of *Pseudomonas aeruginosa*, and its killing concentration against albicans was 62.5 μg/ml (Zhao, 1983). The average value of the minimum bactericidal concentration measured by the Baijiang Dan is between one over ten thousand and one over one million, so that Baijiang Dan is a broad-spectrum bactericidal medicine with strong bactericidal effect. The main chemical components of Baijiang Dan are mercuric chloride (Hg_2_Cl_2_) and calomel (Hg_2_Cl_2_). Due to different formulations, different refining methods, and different experimental methods, the bactericidal efficacy is different. Mercury ions are known to bind to protein and the biocidal effect of the elixir is reduced in the presence of organisms from protein (Su et al., 1991).

#### Hongsheng Dan (Pill)

Hongsheng Dan (Zhao, 1983), with the effects of detoxicating, discharging pus, removing the decay, and promoting tissue regeneration, is an important drug in the treatment of ulcer disease in TCM surgery. B. J. Zhou’s experiment also proved that the elixir had a strong bactericidal effect on common suppurative bacteria, such as *Staphylococcus aureus* and *Escherichia coli in vitro*, and its bactericidal effect was more than 100 times larger than the strong disinfectant carbolic acid. The bactericidal mechanism of Hongsheng Dan is the same as that of other mercury drugs, that is, mercury ions combine with the SH group of bacterial enzymes to inactivate the enzymes and cause bacterial death. However, in the presence of protein organics, mercury ions combined with protein to reduce the bactericidal effect. For example, the minimum bactericidal concentration of Hongsheng Dan for *E. coli* was 10–5. However, when 5% rabbit serum was added into the broth, the concentration of the drug increased by 3 times (3 × 10–5), but it still had no bactericidal effect. Li Q. et al. (2016) believed that while promoting the exfoliation of surrounding dead tissues, Hongsheng Dan also regulated the local growth factor content of wounds, significantly increase the content of IL-2R, IL-6, and TNF, promoted cell mitosis and accelerated wound healing. Yao and Xu (2001) found in their research that Hongshengdan could increase the content of light proline and the number of fibroblasts in wound granulation tissue, promote inflammatory cell infiltration, and enhance the bactericidal effect. Hongshengdan not only could remove wound necrotic tissue, but also could promote wound granulation growth. Wei et al. (1990) studied the effect of Qufu Shengji Powder containing Hongshengdan on the repair of skin ulcers and found that the number of fibroblasts in the treatment group was increased as compared with that in the control group. The dressing changed with Vaseline gauze was active in function and showed an enlarged cell body. It could be seen that the rough endoplasmic reticulum of fibroblasts was developed. Free ribosomes were rich, and the slender collagen fibers in the mesenchyme confirmed the role of Qufu Shengji San in promoting wound healing (Yao and Xu, 2001).

#### Angong Niuhuang Pills (Pill)

Angong Niuhuang pills have the effects of anti-inflammation, improving brain circulation, protecting brain cells, and reducing the damage and cerebral edema caused by the injury. Angong Niuhuang pills are effective for traumatic brain injury, cerebral hemorrhage, ischemia-reperfusion brain injury, and infection-related fever and encephalomyelitis, possibly by inhibiting pro-inflammatory cytokines (Lu et al., 2010). For patients who took Angong Niuhuang pills for 30 days, the serum INF-γ level was significantly reduced, while the IL-4 level was increased, resulting in the reduction of INF-γ/IL-4 ratio, indicating that the Th1/Th2 immune regulation might be related to the therapeutic effect (Liu et al., 2017). Yin (2011) revealed that Angong Niuhuang pills could effectively reduce the expression of Tumor Necrosis Factor-α (TNF-α) after cerebral hemorrhage in rats. In addition, Angong Niuhuang pills could inhibit the inflammatory injury after cerebral hemorrhage by promoting the secretion of anti-inflammatory factor IL -10 and reducing the expression of TNF-α in cerebral ischemic rat. Angong Niuhuang pills can effectively increase the expression of heat shock protein 70 (HSP70) in the process of ischemic brain injury, and thus play a protective role on the damaged nerve cells (Liu et al., 2011). Angong Niuhuang pills may inhibit Bax/Bcl-2 ratio and Caspase-3, and effectively fight against cerebral ischemia-induced brain injury and inhibit apoptosis. Wang G. Z. et al. (2012) showed that Angong Niuhuang pills could increase the expression of phosphorylated Akt in neurons after ischemia and inhibit the occurrence of apoptosis, thereby reducing brain tissue damage and improving neurological function. The protective effect of musk in Angong Niuhuang pills on cerebral ischemic injury may be related to the inhibition of excitatory amino acid toxicity, the down-regulation of the expression of brain tissue matrix metalloproteinase -9, the reduction of the permeability of the blood-brain barrier, the alleviation of the damage to the blood-brain barrier structure, and the alleviation of brain tissue edema after cerebral ischemia (Ni et al., 2012). Liu et al. (2011) reported that Angong Niuhuang pills could also significantly improve blood viscosity, platelet aggregation, and erythrocyte aggregation of rats with cerebral ischemia. Erythrocyte deformability was significantly increased.

#### Shengji Yuhong Gao (Paste)

Shengji Yuhong Gao can promote wound treatment by regulating the level of growth factors, improving wound microcirculation, and reducing the pH value of damaged tissues. It has been widely used for the clinical treatment of wound healing and has achieved satisfactory results in the treatment of gastrointestinal diseases and other diseases (Dong and Liu, 2015). Feng et al. (2008) randomly divided 64 patients with nasal fistula into treatment group and control group. The patients in the treatment group were placed for 7 days, and the dressing was changed with Shengji Yuhong Gao gauze, while the dressing was changed with Vaseline gauze as little as possible. The results showed that Shengji Yuhong Gao could significantly reduce the wound pH of prostate cancer. Yao and Pan (1999) observed the effect of external application of Shengji Yuhong Gao combined with sitz bath with heat-clearing traditional Chinese medicine on the pH of the wound surface at the beginning of anal fissure, and the results showed that compared with the external application of vaseline gauze, the pH of the wound surface in the treatment group was significantly reduced after 7 and 14 days of medication. The results of Zhang et al. (2019a) showed that Shengji Yuhong Gao could significantly increase the level of basic fibroblast growth factor (b-FGF), thereby promoting the synthesis of wound collagen and epithelial growth. In the shaping stage, it also reduces the level of b- FGF, and promotes the degradation of hyperplastic collagen to make it arranged orderly. One of the mechanisms of the effect of Shengji Yuhong Gao on wound repair is mediated by b-FGF. Kou and Wang (2000) demonstrated that the PGF12 content in wound granulation tissue of patients in the treatment group was increased, while the TXB2 content was decreased so that the blood vessels in the granulation tissue were expanded, the blood circulation was accelerated, and the tissue oxygen supply increased, all of which were conducive to wound repair and healing.

#### Zhuhong Gao (Paste)

Zhuhong Gao composed of Cinnabar and red powder has the effects of promoting pus discharge, eliminating slough, promoting tissue regeneration, and healing sore. It can improve wound microcirculation and antibacterial effect, mobilize the body’s antioxidant defense system, and promote wound healing by affecting matrix metalloproteinase (Wang et al., 2016). It is used to treat carrion or no granulation growth on the ulcer surface, or high process of the ulcer mouth, or hot pain with swelling and empyema around the ulcer, or dark ulcer surface and rare empyema (Yan et al., 2013). Zhuhong Gao was an external preparation of traditional Chinese medicine containing mercury, and the average value of mercury content was 16.72% by titration. The research conducted by Lv et al. (2003) showed that Zhuhong Gao had relatively strong effects of removing slough and promoting the growth of tissue, improving wound microcirculation and inhibiting bacteria, and promoting the proliferation and migration of wound keratinocytes and endothelial cells (Mao and Li, 2003). The research conducted by Wang L. et al. (2012) showed that the external application of Zhu Gong Gao could mobilize the antioxidant defense system of the body and improve the antioxidant capacity. With the increase of dose, there was a significant inhibition of the activities of certain antioxidant substances. The research conducted by Lin et al. (2013) indicated that Zhuhong Gao promoted wound healing through its effect on matrix metalloproteinases. At high concentrations, vermouth extract inhibited both MMP-2 and MMP-9 of wound exudate, and the inhibition was enhanced in a context-dependent manner. However, at a low concentration, vermouth extract stimulated the expression of MMP-1 and 2 proteins in normal fibroblasts and enzyme activity *in vitro* but showed an inhibition effect on the expression of MMP-9 in normal fibroblasts.

#### Huafeng Dan (Pill)

The results of Li Q. B. et al. (2013) confirmed that the curative effect of Huafeng Dan combined with western medicine was superior to that of the western medicine-only treatment group. The curative mechanism might be through reducing stroke risk factors Hcy and ET, increasing NO, and reversing vascular endothelial function, thus promoting the recovery of neurological function. The results of the Chen (2013) study showed that the FMA and ADL scores of the Huafeng Dan group were superior to those of the control group (clopidogrel sulfate), indicating that the application of Huafeng Dan had a significant effect on stroke and had few adverse reactions. Zhang et al. (2012) treated with Huafeng Dan could inhibit the overexpression of tumor necrosis factor α (TNF-α), interleukin -1β (IL-1β) and inducible nitric oxide synthase mRNA in microglia induced by LPS, and reduce the contents of TNF-α, IL-1β and nitric oxide in the cell culture supernatant, which further indicated that Huafeng Dan could inhibit the microglia-mediated neuroinflammatory response.

#### Menggen Wusu Shibawei Pills (Pill)

Shi et al. (2015) proved that the Mongolian drug Menggen Wusu Shibawei pills could down-regulate the expression levels of interleukin-2 (IL-2), interleukin-6 (IL-6), and interleukin-8 (IL-8), and significantly improve the symptoms of psoriasis mice. At 1 month, blood mercury levels were slightly increased, indicating a risk of long-term use. Bai et al. (2020) demonstrated that the Mongolian medicine Menggen Wusu Shibawei pills exerted the therapeutic effect on psoriasis by reducing the epidermis thickness and the number of inflammatory cell infiltration in the dermis of the psoriasis-like mouse model, it reduced the spleen index and the cross-sectional area of lymph nodes and intervened in the expression of TH 17-related inflammatory factors. Wang et al. (2020) developed the extraction and separation method of mercury sulfide nanoparticles in Menggen Wusu Shibawei pills. The pharmacodynamic findings are consistent with the bioavailability results of the mercury sulfide nanoparticles, and increased therapeutic effect on adjuvant rheumatoid arthritis rats as the size of the mercury sulfide nanoparticles decreases. Besides, the experimental results of the immune regulation mechanism also show that mercury sulfide nanoparticles have an ideal regulatory effect on rheumatoid arthritis, and are expected to become a new drug for the treatment of rheumatoid arthritis.

#### Zuotai

Jiang et al. (2009) research confirmed, such as Zuotai can reduce due to typhoid Vi polysaccharide. The vaccine caused the body temperature of New Zealand rabbits to increase and xylene caused the ear swelling of mice. The vaccine significantly increased the phagocytic index and clearance index of mice, and significantly prolonged the seizure latency and death latency of mice. The vaccine had significant effects on anticonvulsants, anti-inflammatory, antipyretic and immune enhancement. Zuotai had a certain inhibitory effect on the central nervous system. Studies by Y. Zeng et al. Ceng and He (2005) showed that Zuotai could reduce the spontaneous activity of normal mice, shorten the sleep induction time of mice caused by pentobarbital sodium and chloral hydrate, and prolong the convulsion induction time caused by strychnine and nikethamide. Studies by Li W. Z. et al. (2016) have shown that Zuotai can affect the expression of clock genes, helping to explain the pharmacological and toxicological mechanisms of action of Zuotai. Cell proliferation studies showed that human embryonic kidney cells 293 proliferated significantly at concentrations of 0.25 g·L-1 and 0.125 g·L-1 Zuotai, suggesting that a certain concentration of Zuotai could promote the growth of human embryonic kidney cells. Zuotai promoted the proliferation of 293 cells by inhibiting the apoptosis-related factor Caspase-3.

#### Qishiwei Zhenzhu Pills (Pill)

Qishiwei Zhenzhu pills have a remarkable curative effect on cerebrovascular diseases and can inhibit the formation of cerebral thrombosis. It can reduce the area of cerebral infarction, the water content of the brain, and the pathological changes of brain tissues. Fu et al. (2021) proved that Qishiwei Zhenzhu pills have similar effects with aspirin and compound salvia miltiorrhiza tablets, and the effect is stronger (Jiang, 2010). Liang et al. (2019) demonstrated that Qishiwei Zhenzhu pills had a significant protective effect on the brain tissue of rats with cerebral ischemia-reperfusion injury, which might be related to its inhibition on the increase of blood-brain barrier permeability. Qishiwei Zhenzhu pills could significantly reduce the contents of β-EP, 5-HT, and NA in the brain of rats after cerebral injury, and significantly reduce the Evans Blue content in the brain of ischemic animals, as well as the brain index and brain water content (Hai, 2002). Following the experimental stroke operation, Xu et al. (2020) was observed that rats administered QSW pretreatment had improved neurological function, reduced infarct volume, increased Nissl bodies, improved histopathology, and reduced BBB disruption. It shows that QSW preconditioning has a neuroprotective effect on CI/RI.

Qishiwei Zhenzhu pills significantly inhibited the spontaneous activity of mice, exerted a significant synergistic effect on the sleep dose under the valve of pentobarbital sodium, and exerted a significant synergistic effect on the sleep of mice induced by ether. In a dose-effect relationship, it suggests that the drug has a significant central sedative effect. It has a good improving effect on the learning and memory retrieval loss caused by ethanol in mice and the memory acquisition guarantee caused by anisodine (Wang and Zhou, 2001). Qishiwei Zhenzhu pills have a therapeutic effect on amnesia and hypermnesia caused by cerebrovascular diseases.

Qishiwei Zhenzhu pills did not affect the blood pressure and heart rate of normal rats but had a significant antihypertensive effect on acute renal hypertension (Chen, 1999).

### Toxicology of Mercury and Mercury-Containing Preparations

Exposure to mercury in the general population today comes mainly from three sources: fish consumption, dental amalgam, and vaccines. Each has unique forms of mercury and unique toxicological characteristics and clinical symptoms. Dental amalgam emits mercury vapor that is inhaled and absorbed into the blood (Rocha et al., 2012). Dentists and anyone who uses amalgam fillings are exposed to this form of mercury. Liquid metal mercury (mercury) still enters the home, posing a risk of vapor poisoning and significant cleaning costs. Humans are also exposed to two different but related organic forms, methylmercury (CH_3_Hg^+^) and ethylmercury (CH_3_CH_2_Hg^+^). Fishes are a major source of methylmercury. In nature, mercury is mainly in the form of elemental mercury or sulfides, with a content of about 0.5 parts per million in the earth’s crust. Atmospheric exposure is due to the outgassing of rocks or volcanic activity. Human sources of atmospheric mercury include coal combustion and mining (especially mercury and gold). Elemental mercury in the atmosphere settles in the water where it is converted by microorganisms to organic (methyl or ethyl) mercury, which is ingested by smaller organisms and ultimately consumed by larger fish (Harris, 2003). Large amounts of mercury may accumulate in the tissues of fish at the top of the food chain. The clinical effects of smaller mercury exposures are still controversial. It exists in several forms: inorganic mercury, including metallic mercury, mercury vapor (Hg0), sub mercury (Hg^2+^), and mercury (Hg^+^) salts; Organic mercury, including compounds in which mercury is combined with structures containing carbon atoms (methyl, ethyl, phenyl or the like). The biological behavior, pharmacokinetics, and clinical significance of the various forms of mercury vary by chemical structure. There is some interconversion of the various forms of mercury in the body. Various forms of mercury poison cell function by altering the tertiary and quaternary structure of the protein and by binding to thiols and selenohydryl groups. Therefore, mercury may impair the function of any organ or any subcellular structure. The primary target organ for mercury vapor is the brain (Sun et al., 2021), but peripheral neurological function, renal function, immune function, endocrine and muscular function, and several types of dermatitis have been described (Daume et al., 2021). Aggressive bronchitis and bronchiolitis may lead to respiratory failure with symptoms of the central nervous system such as tremors or essential tremors due to large amounts of acute exposure to mercury vapor (Güngör et al., 2019). Long-term exposure to clinically significant doses of mercury vapor generally leads to neurological dysfunction. Non-specific symptoms such as weakness, fatigue, anorexia, weight loss, and gastrointestinal disorders have been described at low levels of exposure (Sarkar and Bhan, 2005). Chronic poisoning by mercury salts is rare and is usually accompanied by occupational exposure to mercury vapours. Renal toxicity includes tubular necrosis or autoimmune glomerulonephritis, or both (Barne et al., 1976). Immunologic dysfunction includes allergic reactions to mercury exposure, including asthma and dermatitis (De et al., 2007), various types of autoimmunity, as well as inhibition of natural killer cells and destruction of various other lymphocyte subsets. Brain dysfunction is less pronounced than other forms of mercury. Methylmercury reacts with systemic thiols and may therefore interfere with the function of any cellular or subcellular structure (Yuan, 2012). Mercury is thought to interfere with DNA transcription and protein synthesis, including protein synthesis in the developing brain, disrupting endoplasmic reticulum and ribosomal disappearance. There is evidence of damage to many subcellular components in the central nervous system and other organs, as well as mitochondria. Adverse effects on heme synthesis, cell membrane integrity at many sites, free radical production, and neurotransmitter destruction and excitatory nerve stimulation leading to damage to the brain and many parts of the peripheral nervous system are also described (Sakamoto et al., 2018).

#### Cinnabar

Cinnabar is an extremely insoluble compound with a solubility of 1.4 × 10^−2^ g/L. However, some soluble mercury is still present and it also contains free mercury. The contents of free mercury and soluble mercury in Cinnabar were significantly affected by different processing methods (Niu et al., 2015). The research result of soluble mercury and free mercury in Cinnabar by Wang et al. (2013) indicates that the free mercury content of ground Cinnabar is 30–68 pg/g, and the soluble mercury content is 18–38 pg/g. The free mercury content of Cinnabar was 27–354 μg/g and the soluble mercury content was 8–17 μg/g, and it was found that the more times of Cinnabar grinding and washing, the lower the soluble mercury content in Cinnabar. Yang and Tian (1995) reported that no matter where Cinnabar was produced or the processing method adopted, it contained a large amount of soluble and migratory mercury. Particularly, the content of soluble and free mercury in ground Cinnabar was higher than that in grinding and washing Cinnabar. At the same time, they also found that soluble mercury existed in Chinese patent medicines containing Cinnabar.

The results of the acute toxicity test of Cinnabar showed that the LD_50_ of Cinnabar decoction by intravenous injection in mice was 12 g/kg, and the poisoning manifestations in animals were hypokinesia, slow reaction, kidney ischemia, and liver swelling. The results of the subacute toxicity test showed that the mice were orally administrated with 9.5 g/kg Cinnabar for 10–30 days. The histopathological examination showed that the heart, liver, kidney, and other organs showed pathological changes of turbid swelling to different degrees. For the mice given the drug for a long time, a few parts showed eosinophilia. Obvious turbid swelling occurs in the liver, and focal necrosis occurs in severe cases. With the prolongation of drug administration, the damage of renal tissue ranged from mild turbidity to extensive turbidity in renal tubules, the granular cast appeared in renal tubules, and the nuclei of renal tubular epithelium disappeared and showed focal necrosis (Li Q. et al., 2016).

#### Baijiang Dan (Pill)

Chen and Xu (1996) believed that the mechanism of external use of Baijiang Dan for renal injury might be that: after skin trauma, external use of Baijiang Dan in which mercuric chloride was absorbed into the blood and accumulated in the kidney caused the generation of free radicals. Under the action of free radicals, polyunsaturated fatty acids on renal tubular cell membrane enhanced kinase peroxidation, causing cell dysfunction, leading to cell swelling, swelling, degeneration, and eventually tubular necrosis.

According to the reports about the kidney damage caused by Baijiang Dan, it is considered that the mercury toxicity to the kidney is mainly the damage and occlusion of proximal convoluted tubules, followed by the proximal convoluted tubules, and the glomerular damage is milder. Thirty-two mice were treated with external application of 0.4 mg Baijiang Dan every day for 15 consecutive days. The results showed that the renal toxicity was not significant.

According to the dosage conversion between mice and people (according to the body surface area ratio), adults continued to use external application of Baijiang Dan for a long time. It might be relatively safe to use less than 74 mg each time. The animals in this experiment were non-inductive dyed wounds. After the external application of Baijiang Dan, the mercury absorption rate was probably higher than that of infected wounds. The walls of sinuses and ducts tubes were trace tissues. When the external application of Baijiang Dan was applied, the mercury absorption rate was slower and the absorption amount was less. Therefore, the dose of Baijiang Dan for external application could be timely and relaxed, but it could not be used too much (Huang, 2011).

#### Hong Sheng Dan (Pill)

The main component of Hongsheng Dan is mercury oxide, and pharmacological studies have shown that its active component is also Hg^2+^. Low doses of mercury have been shown to have toxic effects on multiple systems, including the urinary, nervous, digestive, immune, reproductive, and cardiovascular systems. The damage to the kidney caused by inorganic mercury was particularly rapid, and the proximal convoluted tubules could become degenerative after 1-h exposure to HgCl_2_ 100 mg/kg in mice. Some researchers observed that the urine microglobulin and serum protein of patients with renal insufficiency were increased and decreased, which are indicators of renal tubular injury, including proximal and distal convoluted tubules and pulp climbing injury. R.M. Chen (1995) observed uniform pathological changes under light and electron microscopes, namely, the damage to the kidney of Hongsheng Dan Preparation was obvious in the distal convoluted tubule, followed by the proximal convoluted tubule, and the damage to glomerulus was milder.

Liu et al. (1986) tested the acute toxicity in mice and found that the median lethal dose was 120.98 ± 1.71 mg/kg. The cumulative toxicity test showed that the toxicity of Hongsheng Dan was cumulative. According to the classification of acute toxicity, Hongsheng Dan is a drug with moderate toxicity after oral administration. Liu et al. (1986) used Qufu Shengji Powder (60% Hongsheng Dan and 10% calomel) as a mercury-containing preparation for external use on the wounds of 51 patients with body surface ulcer. The results showed that the urine mercury values of the groups with different doses of external use mercury-containing preparations all exceeded the standards of Tianjin, China, and had a very significant difference from the blank control group. Moreover, the urine mercury discharge had a significant difference due to the difference in the time of using the mercury-containing preparation.

Chen (1995) pointed out that the use of Hongsheng Dan and mercury-containing preparations could increase the content of free radical MDA in the kidney. Polyunsaturated fatty acids on renal tubular cell membrane enhance lipid peroxidation under the action of free radicals, causing cell dysfunction, cell swelling, and deformation, and finally leading to tubular necrosis. These results indicated that the toxic mechanism of Hongsheng Dan might be related to its subsequent enhancement of renal lipid peroxidation.

#### Angong Niuhuang Pills (Pill)

To evaluate the potential toxicity of Angong Niuhuang pills, *in vitro* and *in vivo* experimental studies were conducted. The cytotoxic concentrations that killed 50% of the cells in five different brain and hepatocyte lines incubated for 48 h with Angong Niuhuang pills, Hg, and/or As compounds showed significant differences: the toxicity of AGNH extracts was much lower than that of MeHg (1/300), mercuric chloride (1/30), As^5+^ (1/25), and As^3+^ (1/50) (Wang et al., 2021). Cinnabar and realgar on cultured hepatocytes and brain cells cytotoxicity are much smaller than the above chemicals. Wu et al. (2011) compared the toxicity of Angong Niuhuang pills, cinnabar and realgar with six common mercury or arsenic compounds *in vitro* using 5 cells including normal rat glial HAPI cells, normal rat liver TRL1215 cells, human liver cancer Hepi2 and Hep3B cells, and human nasopharyngeal carcinoma FaDu cells. The results showed that the toxicity of different mercury and arsenic compounds was different. For example, the cytotoxicity of Cinnabar was only 1/5000 of that of methylmercury, and that of realgar was only 1/10–1/20 of that of arsenic. The toxicity of Angong Niuhuang pills was greater than that of cinnabar and lower than that of realgar.

Sui et al. (2015) gavaged the mice with Angong Niuhuang pills, cinnabar, realgar, mercuric chloride (equivalent to 1/10 of the mercury content of cinnabar), methylmercury (equivalent to 1/100 of the mercury content of cinnabar), sodium arsenite (equivalent to 1/100 of the arsenic content of realgar) and sodium arsenate (equivalent to 1/50 of the arsenic content of realgar) for six consecutive weeks. The results showed that methylmercury and mercuric chloride had the most severe pathological damage to the kidney. The sodium arsenite and sodium arsenite were moderate, and the renal damage caused by realgar was relatively mild. However, cinnabar and Angong Niuhuang pills hardly caused significant pathological changes. Besides, methylmercury and mercuric chloride caused a large amount of mercury accumulation in the kidney. In addition, methylmercury, mercuric chloride, sodium arsenite, and sodium acetate also induced the expression of kidney toxicity-related genes (HO-1, etc.), while Angong Niuhuang pills, cinnabar, and realgar had no effect.

Lu et al. (2011a) gavaged the mice with cinnabar (300 mg/kg), which contained Angong Niuhuang pills, for 4 days for a liver toxicity test, which was compared with methylmercury (MeHg, 2.6 mg/kg) and mercuric chloride (HgCl_2_, 32 mg/kg). Serum transaminases were increased only in the MeHg and HgCl2 groups. Histopathology showed that the liver injury in the MeHg and HgCl_2_ treated mice was more serious than that in the cinnabar and Angong Niuhuang pills groups. It indicated that the liver toxicity of methylmercury and mercuric chloride to mice was much greater than that of cinnabar and Angong Niuhuang pills.

#### Shengji Yuhong Cao (Paste)

The research of Zhang et al. (2016) showed that the low dose (0.5 g/kg) and high dose (1.5 g/kg) of myogenic jade red collagen had no effect on the activity and hair of damaged or intact skin experimental animals, and no bodyweight reduction was observed. The skin structure and inflammatory cell infiltration of the experimental group and the control group were similar. The guinea pig sensitization experiment showed that the bodyweight of the myogenic jade red collagen group was significantly increased compared with the positive control group. The myogenic jade red collagen did not produce a sensitization reaction, and the score and sensitization rate was 0. The guinea pig skin irritation test showed that myogenic jade red collagen had no irritation to the damaged and intact skin structure, telangiectasia, and inflammatory cell infiltration. In conclusion, the myogenic jade red collagen has no skin toxicity, allergy, or skin irritation reaction, and is safe for clinical use.

Liu and Wang (2011) have used Shengji Yuhong Gao for the treatment of anorectal and skin diseases for more than 20 years. One case of the adverse reaction occurred during clinical use. No case report of allergic drug eruption was found from the literature review. This case may be caused by individual differences.

#### Zhuhong Gao (Paste)

The main active ingredient of Zhuhong Gao is Cinnabar, which contains a certain amount of mercury sulfide (HgS). HgS has a certain accumulation in the kidney of the body and can cause inflammatory changes in the skin and the proximal convoluted tubule of the kidney. Tian et al. (2019) studied the mercury accumulation in rat liver, kidney, and brain as well as the toxic effects on liver and kidney after 4 weeks of administration of vermilion cream. The results showed that the biochemical indicators of the enrolled low-dose group were unchanged. The medium and high doses of vermilion cream damaged the structure of the hepatic lobule and the radial arrangement of monolayers of hepatic cords, making the tubular lumen expand, the glomerulus atrophy, and even the glomerulus deficiency. The low dose of vermilion cream had no significant effect on the tissue morphology of the liver and slightly changed the tissue morphology of the kidney. Zhuhong Gao is safe to use at low doses for 1 month, but long-term heavy use will cause mercury accumulation in the liver, kidney, and brain, and lead to hepatorenal toxicity. Guo et al. (2010) study showed that the turbulences of proximal tubule epithelium in animals of high dose group might be related to HgS, and no systemic or local toxic reaction was observed in rabbits of low dose (3.0 mg/kg).

The results of the study by Zhang et al. (2010) showed that a certain amount of Zhuhong Gao applied internally and externally for a certain period, had no acute toxic reaction on rats and no irritation on the damaged skin of guinea pigs.

Dong et al. (2011) randomly divided 66 male SD rats into the damaged skin group, ulcer model group, vaseline matrix group, and high (38.08 mg/kg), medium (19.04 mg/kg), and low (9.52 mg/kg) dosage groups. The results showed that compared with the ulcer model group, there was no significant difference in urinary NAG activity and the urinary RBP content of rats in each dosage group of Zhuhong Gao, vaseline matrix group, and damaged skin group. The pathological examination results of kidney tissues showed that the pathological changes of kidney tissues tended to worsen with the increase of medication dose. Compared with the skin-damaged group, the pathological changes were more obvious in the high-dose Zhuhong Gao group. Compared with the ulcer model group, no significant differences were found in kidney lesions of each dosage group of Zhuhong Gao, vaseline matrix group, and damaged skin group. It was considered safe to use a medium dose of scarlet cream for 2 weeks, while a high dose of scarlet cream for 2 weeks might cause kidney damage.

#### Huafeng Dan (Pill)

The toxicity of arsenic and mercury depends largely on their chemical forms and their metabolites (Lu et al., 2011b). The toxicity differences of arsenic and mercury compounds with different chemical forms are hundreds of times. For example, HgS in Cinnabar is a typical covalent compound with very small solubility, and the oral absorption rate is lower than 0.2%, while that of mercuric chloride reaches 12.5%. It is due to the different forms of mercury that the absorption amounts are quite different, which determines the significant differences in their toxicity (Fu et al., 2013).

In the Zhu et al. (2014) Huafeng Dan group, renal mercury was accumulated, and the expressions of renal injury biomarker Kim-1 and renal stunning transporter Oatl and Oct2 were all lower than those in the mercuric chloride group. In the mercuric chloride group, the expressions of renal efflux transporters Mate-2k and Mr p 4 were all higher than those in other groups. The experimental results showed that the mercury accumulation in rat kidney and renal toxicity of Huafeng Dan with 10 times mercury content were much smaller than those of HgCl, and the effect on renal transporters was smaller than that of HgCl. The above indicators showed no significant difference in WSHFD0, WSHFD1, WSHFD2, and WSHFD3, indicating that the reduction or removal of Cinnabar had little effect on renal toxicity. Studies by Peng et al. (2012) showed that if Huafeng Dan was given to rats at a clinical equivalent dose for 3 weeks, the subacute toxicity to rats would be much lower than that of mercuric chloride and methylmercury. Mercury chloride induced weight loss in rats and significantly increased mercury accumulation in the liver and kidney. Methylmercury also caused increased mercury accumulation in the liver, kidney, and brain tissues of rats, while there were no significant abnormal changes in animals of Huafeng Dan and Cinnabar groups.

The results of Tian et al. (2014) research indicated that the latencies of rats in HgCl_2_ group on the 2–5 days were significantly longer than those in the normal group, indicating that long-term application of HgCl_2_ could weaken the spatial memory ability of rats. However, the trends in the Huafeng Dan group and NaAsO_2_ group were the same as those in the normal group, there was no significant effect on the spatial memory ability of rats.

#### Menggen Wusu Shibawei Pills (Pill)

The efficacy and toxicity of Menggen Wusu were closely related to its soluble form. Organic mercury has a good affinity with biological molecules and can damage nerve cells in brain tissue. The mechanism by which Menggen Wusu can improve the efficacy and reduce the toxicity of drugs through compatibility and combination in prescriptions has been widely concerned by scholars.

The study of Baole et al. (2014) showed that small dose groups of Menggen Wusu Shibawei pills did not produce drug-related toxic reactions in rats. The medium and high dose groups had a certain effect on the liver and kidney function of rats, but the effect was reversible, and it could be restored to normal after drug discontinuation. And high dose group can cause organic lesions in rat kidneys. This was closely related to the processed Menggen Wusu in Menggen Wusu Shibawei pills. It indicated that 0.92 g/kg Menggen Wusu Shibawei pills were a safe dose for rats by gavage. The research by Tong Haiying et al. (Yan et al., 2015) showed that the toxicity of Menggen Wusu Processed Products and Menggen Wusu Shibawei pills commonly used in clinical practices was much lower than that of mercuric chloride and mercurous chloride after a single dose administration to rats. Another study conducted by Tong et al. (2016) showed that rats’ kidney tissue was damaged after continuous administration of Menggen Wusu Shibawei pills for 30 days, which was related to oxidative damage, and the damage in kidney tissue could be gradually recovered after drug discontinuation.

Qing et al. (2014) also evaluated the clinical medication safety of Menggen Wusu Shibawei pills. The results showed that there was no damage to liver and kidney functions or significant change in routine test values of hematuria before and after medication in 60 patients. Hence, the routine dose of Menggen Wusu Shibawei pills could be considered safe and reliable. The research conducted by Qingyu et al. showed that 36 patients with silvery blood disease could well tolerate the treatment with Menggen Wusu Shibawei pills. All the patients had no obvious adverse reactions, and the vital signs, ECG, hematuria routine, and liver and kidney function were normal. In the clinic, the clinical dose could be selected by referring to the prescription dose of 9–11 capsules.

#### Zuotai

Li et al. (2014) have verified the extremely low toxicity of Zuotai through five aspects of single-dose mercury distribution test, subacute toxicity test, acute toxicity test, long-term dose mercury accumulation toxicity test, and clinical safety observation of its compound preparations. The compound preparations have good clinical safety, and the body has no adverse reactions during clinical doses and dosing cycles. However, the kidneys will be affected when large doses are used for a long time. Zhu et al. (2013) also confirmed that after Zuotai was continuously given at the clinical equivalent dose for 4.5 months, there was no significant effect on the external signs, growth and development, reproductive status, routine blood test, serum biochemical indicators, liver and kidney function, and brain histological structure of the mice, but there was a certain effect on the histological structure of the liver, kidney, and spleen. Yang and Jiang (2004) concluded from their research that the long-term use of Zuotai had no obvious toxic effect on rats, nor obvious effect on general state, growth and development, blood indexes, liver and kidney function, and histological structure of important organs of rats. Xiang et al. (2019) studied the effects of long-term administration of Zuotai on rat kidneys. The rats were divided into high (66.70 mg/kg), medium (33.35 mg/kg) and low (16.68 mg/kg) dosage groups. The rats received Zuotai continuously for 90 days, 180 days, and 30 days after drug discontinuation. Compared with the control group, the rats in each dose of Zuotai administration group had no significant abnormalities in appearance signs, behavioral activities, food intake, and defecation. There were no significant abnormalities in kidney superoxide dismutase (SOD) activity, malondialdehyde (MDA) content, kidney coefficient, or renal histopathological examination (*p* > 0.05), suggesting that long-term use of Zuotai had no significant damage to the kidney of rats. The long-term accumulation experiment of mercury in Zuotai conducted by Wu et al. (2016) showed that after long-term administration of Zuotai, the mercury content in brain tissue of mice was slightly higher than that in the blank group, and it could gradually return to normal level after drug discontinuation. Mercury accumulates in the kidney to a certain extent and is excreted through the kidney after drug withdrawal. The accumulation of mercury in the blood, liver, and spleen was not significant (Wu et al., 2016). The above-mentioned studies confirmed that Zuotai and mercury-containing preparations had high safety and no obvious toxic or side effects on the body, but they accumulated to a certain extent in the kidney after long-term use.

#### Qishiwei Zhenzhu Pills (Pill)

According to reports, the main clinical adverse reactions of Qishiwei Zhenzhu pills were nausea, anorexia, mild diarrhea, and dizziness, and most of the symptoms occurred in the drug dose increment period (Liu and Zhu, 2011). During drug administration, patients’ platelets increased significantly compared with normal people (Cui and Lian, 2018). After drug withdrawal, patients’ basophils and serum creatinine increased significantly compared with normal people. However, all differential changes were within the normal range of human indicators. Through the acute and long-term toxicity test in rats, Suo et al. found that Qishiwei Zhenzhu pills had no adverse effect on the appearance, behavior, activity, and diet of rats, and there was no difference between the results of electron microscopy and light microscopy observation on the body weight, liver, and kidney functional and biochemical indicators of rats and the morphology of major organs and tissues as compared with those of normal rats. Xu et al. (2016) administered the maximum dose (10 g/kg) of Qishiwei Zhenzhu pills suspension to the rats by gavage at one time within 24 h, and no animal death, poisoning symptoms, and macroscopic organ abnormalities were observed. Therefore, the maximum oral dose of Qishiwei Zhenzhu pills to rats was 10 g/kg, 600 times of daily dose for adults, with good safety. However, routine blood tests and blood biochemistry indexes should be monitored during clinical application. In the long-term toxicity test such as P. Hai, it was found that after 90 days of administration, a high dose could cause weight loss in rats and mild pathological changes in kidneys at an early stage, all of which were normal. It indicated that this drug had little toxicity and it was safe to take (Hai, 2002).

### Pharmacokinetics of Mercury and Mercury-Containing Preparations

Elemental mercury is also known as metallic mercury (Hg0) (Bernhoft, 2012). Metallic mercury easily passes the blood-brain barrier and the placenta, where it remains in the fetal brain (Ehnert-Russo and Gelsleichter, 2020). However, metallic mercury readily oxidizes to mercury ions when it enters the bloodstream, although not so rapidly as to prevent substantial (Hitosugi et al., 2019). Mercury is absorbed by the central nervous system while still in metallic form. Metallic mercury is primarily excreted as mercury ions (Nordberg et al., 2015). The excretion half-lives of metallic mercury and mercury ions vary widely, from days to months, depending on the deposition organs and the redox state. The mercury salt in the form of Hg_2_Cl_2_ (calomel) is poorly soluble in water and is not readily absorbed by the intestine, although some parts are thought to oxidize to more readily absorbed forms (Lu et al., 2017). Like metallic mercury, mercury ions in blood attach to thiols on red blood cells, metallothionein, or glutathione, or are suspended in plasma (Carocci et al., 2014). Mercury does not efficiently cross the blood-brain barrier, but it accumulates in large amounts in the placenta, fetal tissue, and amniotic fluid.

Methylmercury is the main source of human mercury exposure and is naturally present in fish and relatively stable. Ethylmercury behaves similarly to methylmercury at the cellular level, but has an excretion half-life of about one-third that of methylmercury. The absorption efficiency of methylmercury vapor is similar to that of metallic mercury vapor (80%). Intestinal absorption of methylmercury in fish is also quite efficient, as is dermal absorption (Björnberg et al., 2005). After entering the blood, methylmercury attaches to thiols, especially those found in cysteine. Methylmercury is deposited throughout the body and blood and body equilibrates approximately 4 days after exposure (Zhang et al., 2019b). The distribution to the peripheral tissues appears to be via one or more transporters, in particular the cysteine transporter, which may be attached to the sulfhydryl group of cysteine. Methylmercury is present in the brain, liver, kidneys, placenta, and fetus, especially in the fetal brain, as well as in the peripheral nerves and bone marrow (Berglund et al., 2005). The deposited methylmercury is slowly demethylated to inorganic mercury (David et al., 1998).

#### Cinnabar

Cinnabar is mainly composed of mercury sulfide (HgS), which may cause mercury poisoning if taken in large amounts or improperly used for a long time. Mercury entering the body is mainly distributed in the liver and kidney, causing liver and kidney damage, and can penetrate the blood-brain barrier and directly damage the central nervous system. Cinnabar also had soluble mercury salt in simulated gastric fluid and Hg^+^ dissolved out as the existing form of oral Cinnabar *in vivo*. To study its absorption process *in vivo*, the results showed that the absorption half-life of mice after a single oral administration of Cinnabar was 0.20 h, and the elimination half-life was 13.35 h. The highest concentration in blood was 2.64 pg/ml at 1.09 h after administration. Mercury in Cinnabar was distributed to different extents in the heart, kidney, liver, spleen, lung, large and small brain, and the content in the kidney was the highest (Zheng et al., 2015). Wang et al. (2018) studied the accumulation of Cinnabar *in vivo* and found that the renal mercury content in the ground Cinnabar group was significantly higher than that in the grinding and washing group.

The results of K. Yang study showed that Cinnabar could reduce the pregnancy rate of mice, and mercury in Cinnabar could enter the fetal rats through the placental barrier. The mercury in Cinnabar could be quickly absorbed by pregnant rabbit, young rabbit and adult rabbit after oral administration. The absorption degree of mercury in Cinnabar in the three groups of animals was in the order of pregnant rabbit > young rabbit > adult rabbit (Hiroshi et al., 2018). The neonatal immune blood mercury concentration was found to be as high as 102.3 pg/L, which was significantly higher than that in the adult rabbit and young rabbit. The determination of mercury content in each organ showed that the placenta had the highest content, and the author believed that mercury in the external sand could enter the fetus through the placenta.

Liu and Jia (1997) reported that mercury in Cinnabar could induce an increase in 8-OH-dG in the kidney, believing that mercury could cause DNA oxidative damage in kidney cells. Toxicology studies confirmed that Hg^+^ in plasma mainly combined with serum albumin to form Hg-albumin complex, and mercury in the complex entered renal cells in three forms: Hg-GSH and Hg-Cys complexes formed after exchange with GSH and Cys in plasma, which could be transported into renal cells. It is that exchanging protein containing hydrophobic groups on renal cell membrane into cells. Mercury-containing filterable albumin enters renal cells through cellular endocytosis.

Li W. Z. et al. (2016) administered Tibetan medicines with equal mercury content such as Zuotai, Cinnabar, mercuric chloride, and 1/10 mercury content of methylmercury for seven successive days to compare their renal toxicity. The results showed renal mercury accumulation of up to 250 μg/g with the strongest renal toxicity. The renal mercury accumulation of methylmercury also reached 30 g/g, and the renal toxicity was significant. In contrast, Zuotai and Cinnabar had a renal mercury accumulation of only 2 μg/g, and resulted in low renal toxicity. Therefore, due to different gastrointestinal absorption of different mercury compounds, the mercury distributed and accumulated in the kidney varies greatly, and the toxicity varies greatly. Therefore, mercury sulfide cannot be compared with mercury chloride and methylmercury.

#### Hong Sheng Dan (Pill)

Hongsheng Dan has the function of anti-corrosion and better effect. However, it also has certain toxic and side effects. Therefore, the ways of reducing its renal toxicity should be explored. As the main target organ of toxicological effect of Hongsheng Dan is kidney, drugs for protecting kidney should be used together when using Shengdan preparations, so using diuretic drugs and drinking more water when using, or using mercury-displacing drugs to promote the excretion of mercury, can reduce the toxicity of Hongsheng Dan and Baijiang Dan preparations on kidney. The blood and urine mercury levels of patients with condyloma acuminatum treated with Hongsheng Dan were monitored in Xie et al. (2008). The drug was 0.05–0.3 g each time and the drug was changed once every other day. One week was considered as a course of treatment. The results showed that the concentrations of blood and urine mercury of patients were not significantly increased and mercury poisoning would not be caused. Xu et al. (2019) treated 31 patients with ulcers with external application of Hongsheng Dan and Baijiang Dan. Although the blood mercury concentration of patients after medication was significantly higher than that before medication, serum ALT, BUN, Cr, LPO, and SOD had no significant difference before and after medication, and no mercury poisoning symptom appeared. For adults, the external use of Shengjiang and Jiangdan preparations for a long time would not cause obvious toxic and side effects if the daily doses of Jiu-yi (LI9), Wu-wu (LI5) and Baijiang (GB) were not more than 1.00, 0.20 and 0.07 g, respectively.

#### Angong Niuhuang Pills (Pill)

The distribution, absorption, and excretion of Cinnabar and realgar in Angong Niuhuang pills were as follows: the peak arrival time in blood was 1 h, mercury was mainly distributed in blood and kidney, and arsenic had the highest concentration in blood (Zhao, 2003).

There was no significant difference in the distribution characteristics of mercury and arsenic in normal and cerebral ischemia model rats. The mercury dissolved in simulated gastric fluid and intestinal fluid was 0.00305 and 0.00004% respectively for grinding and washing, and the arsenic dissolved in simulated gastric fluid and intestinal fluid was 0.03375 and 0.00061% respectively for pickled realgar. After 24 h of administration of pure mercuric sulfide and arsenic sulfide, feces of rats were tested, and more than 90.1950% of mercury and 26.4379% of arsenic were excreted. After 24 h of administration of Angong Niuhuang pills, 82.8309% of mercury and 24.4420% of arsenic were excreted. There was no significant difference in the mercury and arsenic contents in rat feces between 120 h after administration of Angong Niuhuang pills and those after administration of pure mercury sulfide and arsenic sulfide. It indicated that mercury in Angong Niuhuang pills existed as complex in rats, and only a small amount of soluble mercury and arsenic might constitute the active part of pharmacology and toxicology, most of which were components that were neither absorbed *in vivo* nor had any pharmacological activity (Wang and Ye, 2003).

#### Menggen Wusu Shibawei Pills (Pill)

The research conducted by Saren (2014) showed that after rabbits were orally administrated with Menggen Wusu Shibawei pills for 1h, their blood mercury concentrations reached the peak, and the elimination rate was fast within 1–5 h and slow after 5 h. The elimination half-life was greater than 25 h. The absorption half-life was 0.218 h and the maximum blood mercury concentration was 3.58 mg/L. Kinetic parameters showed that self-made mercuric sulfide in Menggen Wusu was slowly excreted *in vivo* after oral absorption, so long-term continuous oral administration of drugs containing self-made mercuric sulfide was prone to accumulation poisoning. Patients with liver and kidney diseases, in particular, should be cautious. Tong et al. (2015) have shown that in patients with psoriasis and eczema in the Menggen Wusu Shibawei pills within a month of time, the content of mercury in the blood was increasing, indicating that the long-term use of Menggen Wusu Shibawei pills there is a certain risk. However, Saren (2014) showed that the mercury content of the Mongolian medicine Menggen Wusu Shibawei pills exceeded the standard. In the case of gavage administration of the normal dose group, the mercury had no significant effect on the health of the mice. Mice basically excreted the mercury in the drug in the form of excreta, without causing harm to the body. In the high-dose gavage administration group, most of the mercury in the drug was also excreted through metabolism. Although part of the mercury in Mongolian medicine was accumulated in blood and liver for a period of time. With the prolongation of time, most of it was excreted without causing harm to the body.

#### Zhuhong Gao (Pill)

Kong and Xu (2017) explored the absorption and excretion processes of mercury ion in human body, and the results showed that the concentrations of blood mercury and urine mercury had the trend of changing with time. The role of the time factor differs from group to group. Medication factor could increase the mercury concentration in hematuria of patients, and the concentration decreased significantly after drug discontinuation. Patients with chronic skin ulcer with the ulcer area less than 60 cm^2^ were treated with Zhuhong Gao outside for 6 weeks. There were different types of fluctuation forms of hematuria mercury with the prolongation of medication time, but its value was still within the safe range. Wang et al. (2018) compared the difference of *in vitro* permeation rate of mercury in scarlet Gao in the skin of rats with different injury degrees. The results showed that the skin with different injury degrees was obtained by different treatment methods. The skin of the normal group was in good condition. The stratum corneum was damaged by sand paper polishing, and the superficial layer of dermis was missing by depilatory. The permeation rate of mercury in the normal group was significantly lower than that of the other two groups. The permeation rate of mercury in the sandpaper group and the depilatory group was basically the same, and there was no significant difference in the cumulative permeation amount of mercury. The stratum corneum is the main obstacle to limit the penetration of mercury in scarlet Gao through the skin, and the absorption amount of mercury in scarlet Gao will not be significantly different due to the different degrees of skin injury.

#### Huafeng Dan (Pill)

Rats were given one-time gavage equivalent amount of Huafeng Dan group, Cinnabar, and mercuric sulfide group, and set up a blank control group (normal saline group) (Tian et al., 2015). Compared with the blank control group, the mercury contents in blood, tissues, and urine of each group were increased after 1 h of dosing, and the mercury content in the blood of the Huafeng Dan group was significantly higher than that of other groups. The mercury content in feces of the Huafeng Dan group was significantly lower than that of the Cinnabar group and mercuric sulfide group at 8, 12, and 24 h. The results showed that Huafeng Dan could promote the entry of mercury into the body, and it was mainly distributed in the liver and kidney tissues in a short period (Xu and Cui, 2019).

#### Zuotai

Compared with the control group, Yang et al. (2016) continuously administered Zuotai for 7 and 21 days, respectively, the serum mercury content was lower, although it was increased, indicating that mercury was less absorbed in rats. Mercury in liver and kidney tissues was significantly increased. However, with the prolongation of treatment with Zuotai, the mercury content in liver tissues was significantly reduced, while the mercury in kidney tissues was increased, indicating that mercury in Zuotai was less distributed in the liver of rats, but accumulated in the kidneys. Minimal amounts of mercury were detected in urine, while significant amounts were detected in feces, suggesting that mercury in Zuotai was primarily excreted in feces.

Here are some results about the effect of Zuotai on drug-metabolizing enzymes. In the X.Y. Li study, Zuotai showed no good regularity or dose-effect relationship concerning the effects on the activity, protein, and mRNA expressions of NAT2 and CYPIA2. The regulation of metal on enzyme activity and expression was related to the concentration of metal ions in the body. Within a certain level range, metal could regulate the enzyme activity and expression, but beyond this range, there was no regulation on the enzyme activity and expression. The Li et al. (2010) study showed that after 7 and 21 days of continuous administration of Zuotai, the absorption of crocin -1 was significantly increased and the clearance was significantly decreased after Zuotai administration. Crocin-1 content was significantly lower than that of the control group after administration of Zuotai, suggesting that the *in vivo* distribution of crocin -1 was also affected.

#### Qishiwei Zhenzhu Pills (Pill)

The research results of Li and Suo (2003) showed that the mineral elements in Qishiwei Zhenzhu pills were mainly metabolized by metabolic pathways, suggesting that after long-term administration of Qishiwei Zhenzhu pills, the mineral elements therein would not accumulate in the body, resulting in the toxic effects of heavy metal elements. Xu et al. (2019) used microwave digestion-atomic absorption spectrometry to determine the content of five heavy metal elements including copper, arsenic, cadmium, lead, and mercury in ten batches of Qishiwei Zhenzhu pills. The results showed that the average content of metal elements in the 10 batches of Qishiwei Zhenzhu Pill was from high to low as Hg > Cu > Pb > As > CD, and the mass fraction was higher than 10 μg/kg. The mass fraction of Hg and Cu is higher than 10 mg kg^−1^. The mass fraction of Cd was the smallest, about 20 μg/kg. The content of heavy metals, especially mercury, was higher in seventy kinds of pearls.

## Discussion

Mercury has historically had important medical uses in various countries. The application of mercury-containing preparations and drugs in various countries has a long history and rich experience in clinical practice. With the update of modern research methods, people’s pharmacology and toxicology research on mineral drugs is more in-depth. This article selects various representative mercury-containing preparations in Chinese traditional medicine for external using and oral using, and systematically summarizes their pharmacology, toxicology, and clinical applications. Mercury-containing medicinal materials are usually composed of compound prescriptions, which is also the long-term accumulation of clinical drug experience. Mercury-containing preparations play an irreplaceable role in the treatment of surgical diseases. Representatives of external preparations include Cinnabar, Hongsheng Dan, Baijiang Dan, Zhuhong Gao, Shengji Yuhong Gao, etc. Most of the mercury-containing mineral medicines have the ability to regulate growth factors. It also has pharmacological effects such as antibacterial, anti-inflammatory, anti-corruption, and promoting tissue repair and regeneration. It can also relieve the microcirculation stasis state of the sore surface, promote the proliferation, migration of keratinocytes, endothelial cells, regulate the content of local growth factors, and repair the sore surface (Wang et al., 2017). In the clinical aspect, mercury topical preparations are used to treat postoperative wounds of anal fistula, skin ulcers, and treatment of diabetic foot. Oral mercury-containing preparations include Qishiwei Zhenzhu pills and Zuotai of Tibetan medicine, Huafeng Dan, Angong Niuhuang pills of traditional Chinese medicine. Drugs have pharmacological effects such as anti-inflammatory, sedative and antipyretic, anti-ventilation, anti-convulsant, anti-epileptic, etc., in the nervous system, and anti-hypertension, anti-angina, and anti-atherosclerosis in the cardiovascular and cerebrovascular systems. It is clinically used to treat cardiovascular and cerebrovascular diseases and neurological diseases, such as stroke, cerebral ischemia, brain injury, epilepsy, etc. Mercury-containing preparations such as Zuotai, Qishiwei Zhenzhu pills, and Angong Niuhuang pills are safe for clinical application within a certain range. Improper, excessive or prolonged use may cause acute or chronic toxicity.

At present, the literature reports on mercury-containing preparations and their contemporary preparations were rarely considered from a holistic and in-depth perspective, and mainly focus on general literature, chemical composition, pharmacology, toxicity and clinical observation and so on, but there are few reports on the mechanism of their application in traditional medicine. Due to the lack of literature on Arab medicine and Indian medicine, there is currently a lack of comparative research on mercury-containing preparations in traditional Chinese and foreign medicines, and analyze the pharmacology or toxicity of other medicinal materials in the preparations on mercury.

Most of the mercury in the atmosphere is elemental mercury vapor, which circulates in the atmosphere for up to a year. Most forms of mercury in water, soil, sediment or flora and fauna are inorganic mercury salts and organic mercury. The amount of mercury compounds in organics can be significant (dimethylmercury, phenylmercury, ethylmercury, methylmercury), but the most common organic mercury compound in the environment is methylmercury. The accumulation of methylmercury in marine fish and seafood poses a threat to human health (Li and Suo, 2003). Human exposure in occupational settings is primarily dental amalgam, and the health risks of methylmercury in fish-eating tissues have been the subject of intense debate during several pandemics. Thimerosal, a preservative added to some vaccines, is a public health concern at the source of mercury. In addition, sources of exposure have been added to the use of mercury in cosmetics and religious materials. The EPA (United States Environmental Protection Agency) also states that how a person’s health is affected by exposure to mercury depends on many factors, such as the form of mercury (for example, methyl mercury or elemental (metallic) mercury), the amount of mercury in the exposure, and the age of the person exposed (unborn infants are the most vulnerable), how long the exposure lasts, how the person is exposed (breathing, eating, skin contact, etc.), and the health of those exposed. Depending on the factors listed above, the effects of mercury exposure may be severe, subtle, or may not occur at all. Therefore, making people aware of the health effects of mercury and its sources on the environment can strategically take steps to minimize use and exposure (United States Environmental Protection Agency, 2021). In addition, the residence time of mercury in various tissues, body fluids and excrement in the body is different, and the effect of mercury on various tissues, body fluids and excrement is also different. Mercury levels in blood, hair, and urine reflect recent exposure and are not associated with overall body burden. Blood and urine levels correlated well with each other but not with overall body burden. Since the half-life of all mercury reservoirs in blood is estimated to be between 3 and 5 days, during which excretion or deposition in solid organs occurs, more accurate methods are needed to estimate body burden.

In conclusion, mercury (Hg) is a global pollutant and a well-known neurotoxin that has caused great health concerns. But, on the other hand, mercury-containing minerals have great medicinal value since ancient times. So how to find a balance between mercury as a toxic pollutant and medicinal materials is an urgent matter to be studied and solved, which may require joint efforts in management and scientific research. On the one hand, further reductions in mercury emissions and prevention strategies for mercury risk assessment and management are needed. On the other hand, in addition to studying the accumulation of mercury in the body and tissues, the types and forms of mercury-containing toxic components entering the body through different routes should also be studied, such as the valence and compounds of mercury, and the dose and duration of medication need to be considered, in order to provide reasonable clinical use of mercury-containing mineral medicines and safety reference.

## Data Availability

The original contributions presented in the study are included in the article/Supplementary Material, further inquiries can be directed to the corresponding author.
